# Robertson’s American Tooth Paste

**Published:** 1863-01

**Authors:** 


### ROBERTSON'S AMERICAN TOOTH PASTE.

THIS dentifrice needs no notice except to say that the "Robertson" of its title is ABR. ROBERTSON, M. D., Dentist, Wheeling, Virginia.

